# Comparative Assessment of Fasting and Stimulated C-peptide Levels as Discriminatory Markers of Coronary Artery Disease in Type 2 Diabetes Mellitus: A Cross-Sectional Study

**DOI:** 10.7759/cureus.113029

**Published:** 2026-07-20

**Authors:** Sarosh Kumar, Balakrishnan Valliyot

**Affiliations:** 1 Department of Internal Medicine, Government Medical College, Kannur, IND

**Keywords:** coronary artery disease, fasting c-peptide, insulin resistance, stimulated c-peptide, type 2 diabetes mellitus

## Abstract

Background: Type 2 diabetes mellitus (T2DM) significantly increases the risk of coronary artery disease (CAD), a leading cause of mortality in this population. Early identification of high-risk individuals using reliable biomarkers is crucial for effective prevention. Previous studies have reported that elevated serum C-peptide has been associated with an increased risk of CAD. C-peptide can be assessed under both fasting and stimulated conditions; however, the relative discriminative ability of these measures to identify CAD remains uncertain. This study was therefore undertaken to evaluate the association of fasting and stimulated C-peptide (SCP) levels with the presence of CAD in patients with T2DM and to compare their relative performance in identifying CAD.

Materials and methods: This cross-sectional study was conducted at Government Medical College, Kannur, Kerala, India. It included patients with T2DM of five to 15 years' duration. Patients receiving insulin therapy, with chronic liver disease, an estimated glomerular filtration rate (eGFR) < 45 ml/min/1.73 m², or critical illness were excluded. Clinical, anthropometric, and biochemical parameters, including fasting C-peptide (FCP) and SCP, were assessed. Blood samples for FCP were obtained following a minimum of eight hours of overnight fasting, while SCP samples were collected 90 minutes after administration of a standardized liquid meal. CAD was identified from prior medical records or diagnostic evaluations, with screening performed in those without known CAD. Data were analyzed using IBM SPSS Statistics software, version 24 (IBM Corp., Armonk, NY, USA). Group comparisons were performed using independent-samples t-tests, while logistic regression was employed to identify factors associated with CAD. Receiver operating characteristic (ROC) curve analysis was used to find the optimal C-peptide cut-off values for identifying CAD. A p-value ≤ 0.05 was considered statistically significant.

Results: A total of 282 patients with T2DM (148 males, 134 females; mean age 53.9 ± 7.3 years; mean diabetes duration 8.7 ± 1.9 years) were included. CAD was present in 65 patients (23.1%), comprising 44 with angiographically confirmed disease and 21 with myocardial infarction (four patients with ST-segment elevation myocardial infarction (STEMI) and 17 patients with non-ST-segment elevation myocardial infarction (NSTEMI)). Patients with CAD had significantly higher FCP levels (2.30 ± 0.77 vs. 1.84 ± 0.59 ng/mL; t = 5.04, p < 0.001) and SCP levels (5.24 ± 1.39 vs. 4.04 ± 1.16 ng/mL; t = 6.96, p < 0.001) compared to those without CAD. Logistic regression analysis demonstrated that SCP showed a stronger independent association with CAD, with each unit increase corresponding to nearly a twofold increase in odds. Receiver operating characteristic (ROC) curve analysis showed moderate diagnostic performance for FCP (cut-off ≥ 2.15 ng/mL: sensitivity 64.6%, specificity 72.4%, accuracy 70.6%), whereas SCP exhibited superior discriminatory ability (cut-off ≥ 4.91 ng/mL: sensitivity 73.8%, specificity 84.3%, accuracy 81.8%).

Conclusions: In patients with T2DM, elevated FCP and SCP levels are significantly associated with CAD, with SCP demonstrating a stronger association and superior discriminative ability. These findings highlight its potential as a useful biomarker for earlier identification of high-risk individuals, although confirmation in multicenter prospective studies is warranted.

## Introduction

Type 2 diabetes mellitus (T2DM) represents a significant global public health challenge owing to its substantial morbidity and mortality. Coronary artery disease (CAD) is a common complication and remains a leading cause of mortality in this group. The incidence of both T2DM and CAD is increasing in India largely due to changing lifestyle patterns [1]. T2DM is characterized by relative insulin deficiency resulting from insulin resistance and impaired beta-cell function [2]. Among these, insulin resistance is thought to play an important role in the pathogenesis and progression of macrovascular complications like CAD in patients with T2DM [3].

Many individuals experience prolonged insulin resistance and features of metabolic syndrome long before meeting the diagnostic criteria for T2DM. These metabolic abnormalities contribute to and accelerate atherosclerosis, increasing the risk of CAD in patients with T2DM. A subset of patients with T2DM develops CAD relatively early after diagnosis [4-7]. Early identification of individuals with diabetes who are at increased risk for CAD is crucial for its prevention.

C-peptide, secreted in equimolar amounts with insulin, has emerged as a promising biomarker, as it provides a reliable assessment of endogenous insulin secretion and insulin resistance [8]. Identifying a biomarker for high-risk individuals could facilitate timely and targeted preventive strategies, including intensive lifestyle modification, strict glycemic control, and optimized management of other cardiovascular risk factors.

Multiple studies have reported a significant association between elevated serum C-peptide levels and an increased risk of CAD in patients with T2DM [9-11]. C-peptide can be measured under both fasting and stimulated conditions, providing complementary information on basal insulin secretion and β-cell responsiveness to a standardized meal stimulus, thereby offering indirect insights into insulin resistance and pancreatic secretory function. However, the relative discriminative ability of these measures to identify CAD remains uncertain. Against this background, the present study was conducted to evaluate the association of fasting and stimulated C-peptide (SCP) levels with the presence of CAD in patients with T2DM and to compare their relative performance in identifying CAD.

This work was presented as a poster at the International Diabetes Federation (IDF) World Diabetes Congress 2025, held in Bangkok from April 7 to 10, 2025. The poster, titled “Fasting vs. Stimulated C-Peptide: Exploring Their Role in Coronary Artery Disease Risk in Type 2 Diabetes Mellitus,” was presented on April 10, 2025 (Poster No. BA2025L-1852). The abstract of this presentation was published in the conference supplement of Diabetes Research and Clinical Practice (Volume 230, Supplement 1, 112600, December 2025).

## Materials and methods

Study setting and population

This cross-sectional study was conducted at Government Medical College Kannur, Kerala, South India, between January 2021 and December 2023. Consecutive male and female patients with T2DM, diagnosed according to the 2010 American Diabetes Association criteria [2], were enrolled. Patients aged 41-65 years with a duration of diabetes between five and 15 years were included. Patients receiving insulin therapy, those with chronic liver disease, an estimated glomerular filtration rate (eGFR) < 45 ml/min/1.73 m², or critical illness were excluded.

Data collection method

All participants underwent a comprehensive clinical assessment, which included a detailed history of comorbid conditions, documentation of oral antidiabetic medications, and a thorough physical examination. Anthropometric parameters, including height, body weight, and waist circumference, as well as blood pressure, were recorded. Systemic hypertension was defined according to the 2017 American College of Cardiology/American Heart Association (ACC/AHA) Task Force on Clinical Practice Guidelines [12]. Dyslipidemia was defined based on the 2013 ACC/AHA guideline on the treatment of blood cholesterol to reduce atherosclerotic cardiovascular risk in adults [13].

CAD was defined based on the presence of any one of the following: a documented history of myocardial infarction; electrocardiographic evidence suggestive of ischemia accompanied by raised levels of cardiac biomarkers; a positive treadmill test; echocardiographic findings of regional wall motion abnormalities (RWMA); angiographic confirmation of CAD; or a history of coronary revascularization, including coronary artery bypass grafting or percutaneous coronary intervention. Patients without previously established CAD underwent screening with electrocardiography, exercise stress testing, and echocardiography.

eGFR was calculated using the Chronic Kidney Disease Epidemiology Collaboration (CKD-EPI) equation based on age, sex, and serum creatinine [14], and calculations were performed using the National Kidney Foundation eGFR calculator available at: https://www.kidney.org/professionals/gfr_calculator [15]. Following an overnight fast of at least eight hours, blood samples were obtained to measure fasting plasma glucose (FPG), fasting C-peptide (FCP), lipid profile, glycated hemoglobin (HbA1c), blood urea, and serum creatinine.

Serum C-peptide was measured using the Beckman Coulter Access 2 Immunoassay System (Beckman Coulter, Inc., Brea, CA, USA) based on a paramagnetic particle chemiluminescent immunoassay. This one-step sandwich immunoassay utilizes monoclonal anti-C-peptide-coated paramagnetic particles and an alkaline phosphatase-labelled anti-C-peptide conjugate to form an antigen-antibody complex. After magnetic separation and washing, a chemiluminescent substrate is added, and the emitted light, proportional to the C-peptide concentration, is measured using a luminometer. Quantification is performed automatically using stored calibration data. The analytical range for serum samples was approximately 0.01-30.0 ng/mL. All measurements were conducted in accordance with the Clinical and Laboratory Standards Institute (CLSI) EP28-A3c guidelines [16], and serum C-peptide was reported as ng/mL in this study.

For the measurement of SCP, a blood sample was obtained 90 minutes after administration of a standardized liquid meal. The meal was prepared using Ensure Diabetes Care powder (Abbott Healthcare Pvt. Ltd., Mumbai, Maharashtra, India) and administered according to body weight at a dose of 6 kcal/kg, dissolved in 300 mL of water, and consumed within five minutes. As per the nutritional composition, 100 g of the powder provided 435 kcal, comprising 59.79 g of carbohydrates, 20.60 g of protein, and 11.90 g of fat. Carbohydrates constituted the predominant macronutrient, contributing 239.16 kcal (54.98% of total energy), followed by fats providing 107.10 kcal (24.98%) and proteins contributing 82.40 kcal (19.22%). This composition ensured a balanced macronutrient distribution for standardized stimulation.

Ethical approval

The study received ethical approval from the Institutional Ethics Committee (IEC) of Government Medical College, Kannur, Kerala, India (IEC No. 96/2019/GMCK; dated 14/08/2020 and 20/02/2023). All participants included were adequately informed about the study, and written informed consent was obtained before their inclusion.

Statistical analysis

The collected data were entered into Microsoft Excel (Microsoft Corp., Redmond, WA, USA) and subsequently analyzed using IBM SPSS Statistics for Windows, Version 24 (IBM Corp., Armonk, NY, USA). Categorical variables were summarized as frequencies and percentages, and continuous variables as mean ± standard deviation. Group comparisons were performed using Pearson’s chi-square test for categorical variables and an independent-samples t-test for mean serum FCP and SCP levels. Multivariable binary logistic regression was used to identify the factors and estimate their association with CAD. Receiver operating characteristic (ROC) curve analysis was performed to determine the optimal cut-off values of FCP and SCP for identifying CAD. A probability value (p) ≤ 0.05 was considered statistically significant.

## Results

A total of 282 patients with T2DM were enrolled, with 148 males (52.5%) and 134 females (47.5%). The participants had a mean age of 53.9 ± 7.3 years and a mean duration of diabetes of 8.7 ± 1.9 years. Current smoking was reported by 44 individuals (15.6%), while 60 participants (21.3%) consumed alcohol regularly. Comorbid systemic hypertension was observed in 190 patients (67.4%), and dyslipidemia was present in 203 patients (72.0%). Anthropometric assessment showed a mean body mass index (BMI) of 25.32 ± 2.83 kg/m² and an average waist circumference of 90.78 ± 7.73 cm. The mean systolic and diastolic blood pressures were 135.04 ± 11.45 mmHg and 83.90 ± 7.07 mmHg, respectively. The mean FCPe level was 1.94 ± 0.67 ng/mL, while the mean SCP level was 4.32 ± 1.32 ng/mL.

CAD was identified in 65 patients (23.1%) within the study cohort, whereas 217 patients (76.9%) had no evidence of CAD. Among those with CAD, 44 patients (67.7%) had angiographic confirmation of the diagnosis. The remaining cases were diagnosed based on clinical presentation, including four patients (6.1%) with ST-elevation myocardial infarction (STEMI) and 17 patients (26.2%) with non-ST-elevation myocardial infarction (NSTEMI). Among the 44 patients with angiographically confirmed CAD, the distribution of vessel involvement revealed that 29 had triple-vessel disease, nine had double-vessel disease, and six patients had single-vessel disease.

Among patients in the CAD group, 49 (75.4%) were male, and 16 (24.6%) were female, with a significant association between male gender and CAD (χ² = 17.77, p < 0.001; OR = 3.65, 95% CI: 1.96-6.82). With respect to smoking status, 27 (41.54%) were current smokers and 38 (58.46%) were non-smokers; smoking showed a strong association with CAD (χ² = 43.15, p < 0.001; OR 8.36, 95% CI: 4.16-16.82). Systemic hypertension was present in 57 (87.69%) patients and was significantly associated with CAD (χ² = 15.86, p < 0.001; OR 4.50, 95% CI: 2.05-9.90). Similarly, dyslipidemia was observed in 61 (93.85%) patients and was significantly associated with CAD (χ² = 20.02, OR = 8.06, 95% CI: 2.82-23.01, p < 0.001).

The mean age was significantly higher in patients with CAD compared to those without CAD (56.65 ± 6.17 vs 52.98 ± 7.37 years; t = 3.65, p < 0.001). The mean duration of diabetes did not differ significantly between the two groups (8.88 ± 2.04 vs 8.62 ± 1.88 years; t = 0.96, p = 0.339). Similarly, no significant differences were observed in BMI (25.14 ± 2.29 vs 25.38 ± 2.98 kg/m²; t = −0.58, p = 0.562) or waist circumference (91.03 ± 7.22 vs 90.71 ± 7.90 cm; t = 0.29, p = 0.770). There were no significant differences in systolic (134.58 ± 12.09 vs 135.17 ± 11.28 mmHg; t = −0.36, p = 0.718) or diastolic blood pressure (84.62 ± 6.60 vs 83.69 ± 7.21 mmHg; t = 0.93, p = 0.354) between the groups (Table 1). 

**Table 1:** Baseline characteristics of participants with and without CAD CAD: coronary artery disease; DM: diabetes mellitus; BMI: body mass index in kg/m^2^; WC: waist circumference; SBP: systolic blood pressure; DBP: diastolic blood pressure

Parameters	CAD (n - 65)	Non-CAD (n - 217)	t-value	p-value
Age (years)	56.65 ± 6.17	52.98 ± 7.37	3.65	0.001
Duration of DM (years)	8.88 ± 2.04	8.62 ± 1.88	0.96	0.339
BMI (kg/m^2^)	25.14 ± 2.29	25.38 ± 2.98	- 0.58	0.562
WC (cm)	91.03 ± 7.22	90.71 ± 7.90	0.29	0.770
SBP (mm of Hg)	134.58 ± 12.09	135.17 ± 11.28	- 0.36	0.718
DBP (mm of Hg)	84.62 ± 6.60	83.69 ± 7.21	0.93	0.354

Comparison of biochemical parameters revealed no significant differences between CAD and non-CAD groups in fasting plasma glucose (140.74 ± 28.76 vs 145.21 ± 32.96 mg/dL; t = −0.99, p = 0.325), postprandial plasma glucose (224.82 ± 41.10 vs 232.42 ± 46.15 mg/dL; t = −1.19, p = 0.234), or HbA1c (8.29 ± 1.09% vs 8.19 ± 1.29%; t = 0.57, p = 0.448). Lipid profile parameters, including total cholesterol, low-density lipoprotein cholesterol (LDL-C), high-density lipoprotein cholesterol (HDL-C), and triglycerides, were also comparable between the groups. Among renal parameters, blood urea did not differ significantly (27.05 ± 7.55 vs 26.21 ± 5.02 mg/dL; t = 1.04, p = 0.301), whereas serum creatinine (0.99 ± 0.19 vs 0.93 ± 0.17 mg/dL; t = 2.11, p = 0.036) and serum uric acid (6.01 ± 0.87 vs 5.61 ± 1.04 mg/dL; t = 2.84, p = 0.005) were significantly higher in the CAD group. eGFR was similar between the groups (84.38 ± 16.75 vs 85.39 ± 17.58 mL/min/1.73 m²; t = −0.41, p = 0.683) (Table 2).

**Table 2:** Comparison of biochemical profiles between participants with and without CAD. CAD: coronary artery disease; FPG: fasting plasma glucose; PPG: prandial plasma glucose; HbA1C: glycated hemoglobin; TG: triglyceride; HDL-C: high-density lipoprotein cholesterol; LDL-C: low-density lipoprotein cholesterol; VLDL: very low-density lipoprotein cholesterol; Bld. urea: blood urea; eGFR: estimated glomerular filtration rate

Parameters	CAD (n - 65)	Non-CAD (n - 217)	t-value	p - value
FPG (mg/dl)	140.74 ± 28.76	145.21 ± 32.96	-0.99	0.325
PPG (mg/dl)	224.82 ± 41.10	232.42 ± 46.15	-1.19	0.234
HbA1C (%)	8.29 ± 1.09	8.19 ± 1.29	0.57	0.448
Total cholesterol (mg/dl)	195.31 ± 26.64	199.16 ± 24.62	-1.09	0.278
TG (mg/dl)	142.85 ± 57.29	135.79 ± 41.69	1.09	0.276
HDL-C (mg/dl)	46.09 ± 7.78	46.74 ± 7.16	-0.62	0.533
LDL-C (mg/dl)	120.20 ± 22.29	123.84 ± 22.23	-1.16	0.248
Blood urea (mg/dl)	27.05 ± 7.55	26.21 ± 5.02	1.04	0.301
Serum creatinine (mg/dl)	0.99 ± 0.19	0.93 ± 0.17	2.11	0.036
Serum uric acid (mg/dl)	6.01 ± 0.87	5.61 ± 1.04	2.84	0.005
eGFR (mL/min/1.73 m2)	84.38 ± 16.75	85.39 ± 17.58	-0.41	0.683

**Figure 1:**
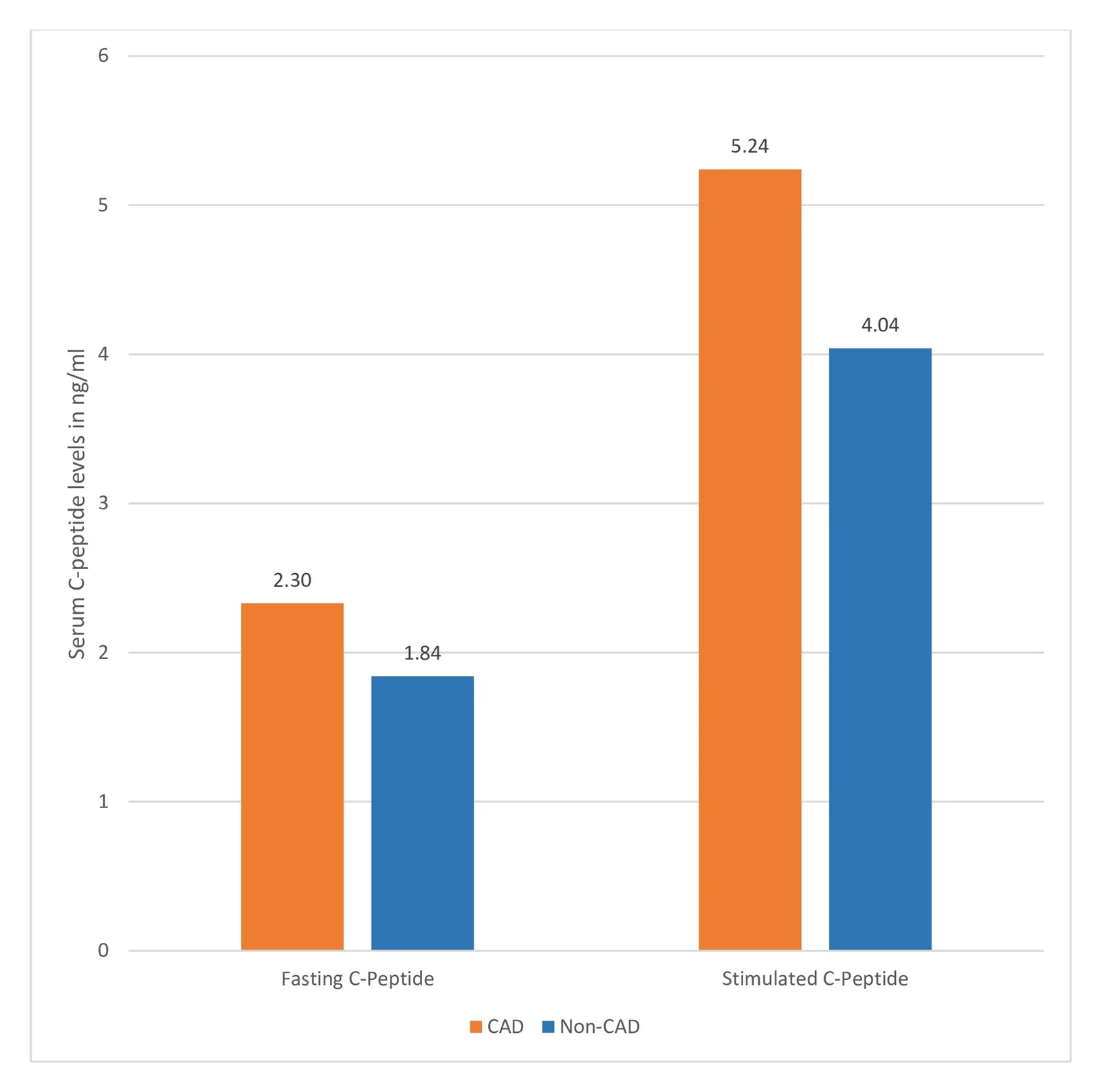
Comparison of fasting and stimulated C-peptide levels between patients with and without CAD Fasting C-peptide levels: CAD, 2.30 ± 0.77 ng/ml vs. non-CAD, 1.84 ± 0.59 ng/ml; t = 5.04, p < 0.001; Stimulated C-peptide levels: CAD, 5.24 ± 1.39 ng/ml vs. non-CAD, 4.04 ± 1.16 ng/ml; t = 6.96, p < 0.001. CAD: coronary artery disease

Patients with CAD demonstrated significantly higher mean FCP levels compared to those without CAD (2.30 ± 0.77 vs. 1.84 ± 0.59 ng/mL), with this difference being statistically significant (t = 5.04, p < 0.001). Similarly, SCP levels were also markedly elevated in the CAD group (5.24 ± 1.39 vs. 4.04 ± 1.16 ng/mL), again showing a highly significant difference (t = 6.96, p < 0.001) (Figure 1).

**Figure 2:**
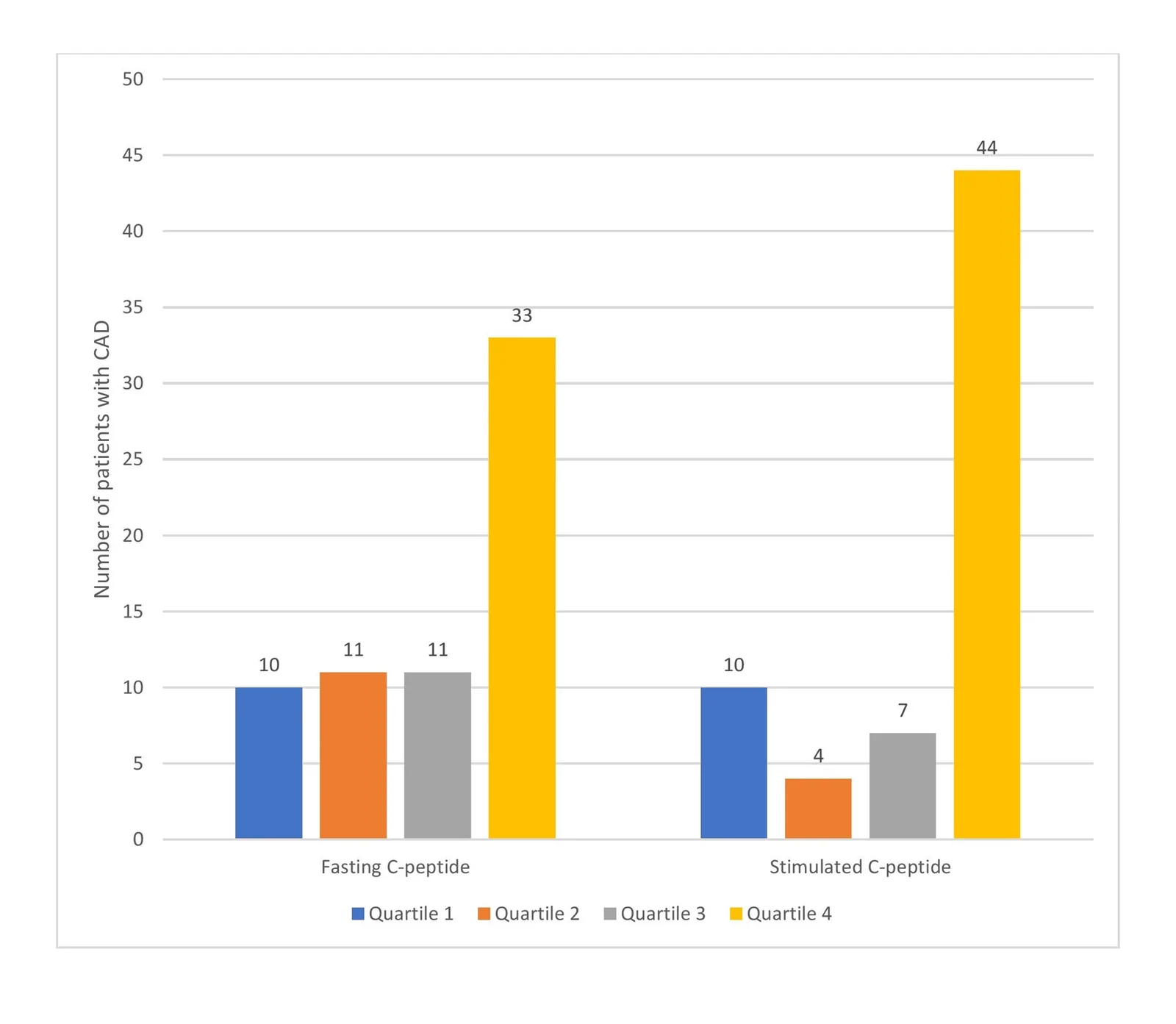
Fasting and stimulated C-peptide quartile-wise distribution of CAD patients CAD cases were distributed across quartiles as follows: Quartile 1 (Q1; fasting ≤1.45: 10 (15.4%); stimulated ≤2.96: 10 (15.4%)), Q2 (fasting 1.46–1.94: 11 (16.9%); stimulated 2.97–4.20: 4 (6.2%)), Q3 (fasting 1.95–2.34: 11 (16.9%); stimulated 4.21–5.19: 7 (10.8%)), and Q4 (fasting >2.35: 33 (50.8%); stimulated >5.19: 44 (67.7%)). Most cases were concentrated in Q4, indicating higher CAD prevalence with increasing C-peptide levels. CAD: coronary artery disease

Patients with CAD were stratified by quartiles of FCP and SCP levels to assess their distribution across increasing ranges. A predominant proportion of CAD cases was observed in the highest quartile (Q4), corresponding to FCP values >2.35 ng/mL (33 patients, 50.8%) and SCP values >5.19 ng/mL (44 patients, 67.7%). In addition, a smaller yet notable fraction of patients was categorized in the third quartile (Q3), with FCP levels between 1.95 and 2.34 ng/mL (11 patients, 16.9%) and SCP levels between 4.21 and 5.19 ng/mL (seven patients, 10.8%). Collectively, these findings demonstrate a clear clustering of CAD cases in the upper quartiles, particularly Q4, indicating a progressive increase in CAD prevalence with rising FCP and SCP concentrations (Figure 2).

Binary multivariable logistic regression analysis determined the independent factors associated with CAD. Dyslipidemia, smoking, and SCP emerged as statistically significant factors associated with the presence of CAD. Individuals with a history of smoking exhibited nearly a 3.7-fold increase in the likelihood of developing CAD compared to non-smokers, and the presence of dyslipidemia was associated with approximately 3.8 times higher odds of CAD. SCP levels showed a strong and independent association with CAD risk. Specifically, each 1 ng/mL increment in SCP was associated with a 95.7% increase in the odds of CAD (B = 0.671, p < 0.001, Exp(B) = 1.957, 95% CI: 1.355-2.826) (Table 3).

**Table 3:** Logistic regression analysis of factors associated with CAD CAD: coronary artery disease; B: coefficient; Wald: Wald statistic; Exp (B): exponentiated B coefficient (odds ratio); CI: confidence interval. Multivariable logistic regression analysis of CAD in patients with T2DM; T2DM: type 2 diabetes mellitus in years, HTN: systemic hypertension; DLP: dyslipidemia; BMI: body mass index; eGFR: estimated glomerular filtration rate; HbA1c: glycated hemoglobin; FCP: serum fasting C-peptide levels; SCP: serum stimulated C-peptide levels.

Variables	B	Wald	Sig	Exp (B)	95 % CI for Exp (B)
Age	0.002	0.005	0.943	1.002	0.937 - 1.072
Male sex	0.680	2.542	0.111	1.974	0.856 - 4.552
Duration of T2DM	-0.003	0.001	0.981	0.997	0.780 - 1.274
HTN	0.957	3.443	0.064	2.604	0.947 - 7.159
DLP	1.338	4.873	0.027	3.811	1.162 - 12.502
Smoker	1.309	7.743	0.005	3.701	1.472 - 9.304
BMI (kg/m^2^)	-0.014	3.475	0.062	0.870	0.751 - 1.007
HbA1c (%)	0.196	1.460	0.227	1.217	0.885 - 1.673
eGFR (ml/min/1.73 m^2^)	-0.002	0.021	0.884	0.998	0.974 - 1.023
FCP (ng/ml)	0.217	0.385	0.535	1.242	0.627 - 2.460
SCP (ng/ml)	0.671	12.805	0.001	1.957	1.355 - 2.826

ROC curve analysis demonstrated that both FCP and SCP were significant discriminators of CAD. FCP showed moderate diagnostic performance with an area under the curve (AUC) of 0.687 (95% CI: 0.605-0.770, p < 0.001); at a cutoff value of ≥ 2.15 ng/mL, it yielded a sensitivity of 64.6% and specificity of 72.4%, with a positive predictive value (PPV) of 41.2%, negative predictive value (NPV) of 87.3%, and an overall accuracy of 70.6%. In comparison, SCP demonstrated superior discriminative ability, with an AUC of 0.762 (95% CI: 0.683-0.840, p < 0.001); using a cutoff of ≥ 4.91 ng/mL, it achieved a sensitivity of 73.8% and specificity of 84.3%, along with a PPV of 58.4%, NPV of 91.5%, and an overall accuracy of 81.8%, indicating better overall diagnostic performance than FCP for identifying CAD.

## Discussion

In this study involving 282 patients with T2DM, CAD was identified in 23.1% of participants, underscoring the substantial burden of macrovascular complications in this population. Established risk factors of CAD, including advancing age, male gender, smoking, dyslipidemia, and systemic hypertension, showed significant associations with CAD, consistent with their well-documented role in accelerating atherosclerosis in individuals with T2DM [17].

A key objective of this research was to compare the discriminative ability of FCP and SCP levels in identifying CAD among patients with T2DM. An important finding was that both FCP and SCP levels were significantly elevated in patients with CAD in comparison to those without CAD. However, beyond this general association, comparative analyses revealed that SCP demonstrated a stronger and more consistent relationship with CAD than FCP. Quartile-based analysis showed a clear clustering of CAD cases in the higher quartiles of both measures, but the gradient of risk was more pronounced with SCP, suggesting superior discriminatory capacity.

Multivariable logistic regression analysis further reinforced this distinction, identifying SCP, along with smoking and dyslipidemia, as an independent factor associated with CAD. Notably, each one-nanogram-per-millilitre increase in SCP levels was associated with an approximately twofold increase in the odds of CAD. ROC curve analysis provided additional support, demonstrating superior discriminative performance of SCP, with higher sensitivity, specificity, and overall diagnostic accuracy at an optimal cutoff value of ≥ 4.91 ng/mL compared with fasting C-peptide.

The observed superiority of SCP may reflect its closer physiological relevance; however, this remains a hypothetical explanation. While FCP represents basal insulin secretion, SCP captures the β-cell response to a glucose challenge and may better reflect postprandial insulin dynamics and glycemic excursions. Given that postprandial hyperglycemia has been implicated as a stronger determinant of macrovascular complications than fasting hyperglycemia-potentially through mechanisms such as oxidative stress, endothelial dysfunction, and accelerated atherogenesis-this may partly account for the findings [18,19]. However, these mechanisms were not directly evaluated in the present study and require confirmation through dedicated future research.

Elevated C-peptide levels reflect underlying insulin resistance, a central mechanism linking T2DM and atherosclerosis. As insulin resistance progresses, compensatory hyperinsulinemia leads to increased C-peptide levels due to its equimolar co-secretion with insulin. The stronger association of SCP with CAD observed in this study suggests that it may better capture the severity of insulin resistance and postprandial metabolic stress compared with FCP levels. Experimental studies have also suggested a direct role of C-peptide in atherogenesis, including stimulation of smooth muscle cell proliferation and inflammatory pathways [20,21].

These findings are consistent with prior studies demonstrating an association between elevated serum C-peptide levels and CAD. Wang et al. demonstrated that elevated FCP levels were independently associated with CAD in patients with T2DM, even after adjustment for conventional risk factors [9]. Similarly, studies from Indian populations have reported significant associations between FCP levels and both the presence and severity of CAD [10]. Furthermore, subgroup analysis of the same cohort demonstrated a significant positive association between FCP levels and CAD in T2DM patients aged 41-60 years [11]. In a large prospective cohort, Cabrera de Leon et al. reported that elevated serum C-peptide levels were associated with an enhanced risk of CAD and myocardial infarction in the general population [22].

Additional findings from this study support the broader cardiometabolic risk profile associated with CAD. Although glycemic parameters such as fasting plasma glucose, postprandial glucose, and HbA1c were comparable between those with and without CAD, serum creatinine and uric acid levels were significantly higher in patients with CAD, suggesting a link between renal dysfunction, metabolic syndrome, and atherosclerosis [23]. Importantly, no significant differences were observed in the use of oral antidiabetic medications, minimizing potential treatment-related confounding.

Most previous studies have relied predominantly on FCP measurements, with limited direct comparison to stimulated levels. The present study adds to the existing literature by demonstrating the superior discriminative performance of SCP in identifying coronary artery disease compared with FCP. Overall, these findings suggest that although both FCP and SCP are associated with CAD, SCP may offer greater clinical utility as a discriminative biomarker for identifying CAD in patients with T2DM.

Strengths and limitations

A key strength of this study is the simultaneous measurement of FCP and SCP in the same cohort, enabling direct comparison of their discriminatory ability for CAD. The use of a standardized stimulation protocol further enhances the reliability and comparability of SCP measurements. In addition, stringent inclusion and exclusion criteria were applied to minimize potential confounding; patients with renal dysfunction (eGFR <45 ml/min/1.73 m²) and critical illness, both of which may spuriously elevate C-peptide levels, were excluded. The study also deliberately restricted inclusion to individuals with five to 15 years of diabetes duration to reduce heterogeneity associated with extremes of β-cell function.

However, the cross-sectional design precludes causal inference and assessment of future risk prediction. The restricted age group and disease duration limit generalizability. CAD ascertainment was heterogeneous, raising the possibility of misclassification bias. Residual confounding, including the effects of antidiabetic therapies, cannot be excluded, and the moderate sample size may lead to model overfitting without external validation. More prospective studies are required to confirm these findings and determine clinical relevance.

## Conclusions

In patients with T2DM, elevated levels of both FCP and SCP are significantly associated with the presence of CAD. Notably, SCP demonstrates a stronger association and superior discriminative ability compared with FCP in identifying CAD. These findings suggest that SCP may serve as a more sensitive and useful biomarker for CAD and may facilitate the earlier identification of high-risk individuals. However, these observations, derived from a cross-sectional study, warrant validation through multicentre prospective studies.
